# Fundamental methodological issues

**DOI:** 10.1038/s41405-023-00158-4

**Published:** 2023-07-24

**Authors:** Chris Longbottom

**Affiliations:** Consultant - Chris Longbottom, Newport-on-Tay, DD68DW United Kingdom

**Keywords:** Health care, Dentistry

As someone who has spent over 35 years in cariology research, with more than 20 of those years investigating carious lesion activity assessment, and as a consultant to a company which has developed a novel technology aimed at differentiating between active and inactive carious lesions, I was particularly interested in the above referenced article by Amaechi et al. in BDJ Open.

A number of methodological issues in the article raise concerns about the validity of the results as reported.

The most significant of these issues is the fact that a single examiner carried out the ICDAS exam, selected the study lesions on the basis of that, then immediately carried out the LC Rinse assessment—in other words, the same person identified and selected the test and control lesions, then immediately carried out the test exam, after that reference exam, i.e. there was no blinding of the test examiner to which lesions were designated in the reference examination as active, inactive or sound, when carrying out the test examination—this leaves the study open to a high and significant risk of examiner bias.

The close proximity in time of the reference and test examinations would only serve to increase the likelihood of such bias.

The fact that the sensitivity, specificity, diagnostic accuracy and Odds Ratio figures of the “LC Rinse” assessment for the “Selected study” surfaces fell significantly when the “Full dentition” surfaces were assessed suggests that there was indeed significant bias present when the “Selected study” sites were assessed.

A related issue of concern is the lack of mention in the article about how exactly the authors defined the “qualitative positive test response” of the LC Rinse signal—the criteria used for designating a positive or negative signal are not provided. There is no mention of “areas of interest” or how the examiner ensured that any increased fluorescence signal exactly corresponded spatially to the specific surface area of each lesion previously identified. This is of particular relevance in relation to figure 1 in the article, in which almost all of the increased yellow fluorescent light signal visible on the three upper incisors in the post-rinse image appears to emanate from curved lines corresponding to the gingival crevices rather than the areas of decalcification visible in the pre-rinse images.

A further issue concerns the distribution of the relative numbers of the separate Occlusal and Free Smooth Surface (FSS) lesions in the Selected Study Teeth samples—this distribution could significantly bias the outcome of the study analysis, since it is relatively easier to visually assess anterior teeth FSS lesions than posterior teeth occlusal lesions. The article does not specify exactly how the “test and control sites were then selected” in terms of occlusal or FSS sites nor does it provide the distribution figures for the occlusal and free smooth surface sites.

This site distribution factor may have also contributed to the dramatic order of magnitude fall in the value between the “Selected study teeth” assessment and “Full dentition” assessment values for the Odds Ratios noted in the article—Table 4.

The issue of examiner bias in gathering data to assess novel technologies for disease assessment is a critical issue in the development of the methodology used in such research. The methodology reported in the Amaechi et al. article appears to have been open to a significant level of examiner bias, which fundamentally undermines the results as presented.

The authors of the original manuscript have been invited to reply.

